# Comparison of the Efficiencies of Buffers Containing Ankaferd and Chitosan on Hemostasis in an Experimental Rat Model with Femoral Artery Bleeding

**DOI:** 10.4274/tjh.2014.0029

**Published:** 2016-02-17

**Authors:** Serkan Abacıoğlu, Kemal Aydın, Fatih Büyükcam, Ural Kaya, Bahattin Işık, Muhammed Evvah Karakılıç

**Affiliations:** 1 Osmaniye State Hospital, Clinic of Emergency, Osmaniye, Turkey; 2 Dışkapı Yıldırım Beyazıt Training and Research Hospital, Clinic of Emergency, Ankara, Turkey; 3 Bülent Ecevit University Faculty of Medicine, Department of Emergency, Zonguldak, Turkey; 4 Keçiören Training and Research Hospital, Clinic of Emergency, Ankara, Turkey; 5 Ankara Numune Training and Research Hospital, Clinic of Emergency, Ankara, Turkey

**Keywords:** Bleeding, Ankaferd, Chitosan, Hemostasis

## Abstract

**Objective:**

In the first assessment of trauma patients with major vascular injuries, we need effective and rapid-acting homeostatic materials. In this study we compare the efficiencies of Ankaferd Blood Stopper® and a chitosan linear polymer (Celox®) in an experimental rat model with femoral artery bleeding.

**Materials and Methods:**

Thirty male Wistar albino rats weighing 200-250 g were divided into 3 groups: control, Ankaferd, and chitosan. The femoral artery and vein were visualized and bleeding was started by an incision. The bleeding time was recorded and categorized as ‘bleeding stopped at the second minute’, ‘bleeding stopped at the fourth minute’, and ‘unsuccessful’ if bleeding continued after the fourth minute.

**Results:**

In the control group, 60% of the bleeding did not stop. In the first 4 min in the Ankaferd group, the bleeding stopped in all rats; only in 1 of the rats in the chitosan group did the bleeding not stop. In stopping the bleeding in the first 4 min, Ankaferd was similar to chitosan but better than the control group; the chitosan group was similar to the control, but the p-value was close to significance.

**Conclusion:**

For major arterial bleeding, the main treatment is surgical bleeding control, but outside of the hospital we can use buffers containing Ankaferd and chitosan on the bleeding region. The results of this study should be supported with larger studies. Furthermore, in our study, healthy rats were used. New studies are needed to evaluate the results of hypovolemic and hypotensive cases with major artery bleeding.

## Abstract (tr)

**Amaç:**

Büyük damar yaralanması olan travma hastalarının ilk müdahalesinde etkin ve hızlı etkili kanama durdurucu malzemelere ihtiyaç duyulmaktadır. Biz bu çalışmamızda, femoral arter kanamalı deneysel fare modelinde Ankaferd Blood Stopper® ve chitosan lineer polymerin (Celox®) etkinliklerini karşılaştırdık.

**Gereç ve Yöntem:**

Ağırlıkları 200-250 gram olan 30 erkek Wistar albino fare, kontrol, Ankaferd ve chitosan olmak üzere üç gruba ayrıldı. Femoral arter ve ven görünür hale getirildi ve bir kesi ile kanama başlatıldı. Kanama zamanı kaydedildi ve ‘kanaması ilk iki dakikada duranlar’, ‘kanaması ilk dört dakikada duranlar’ şeklinde ve eğer dördüncü dakikadan sonra kanama halen devam ediyorsa ‘başarısız’ olarak kaydedildi.

**Bulgular:**

Kontrol grubunda, kanamaların %60’ı durmadı. İlk dört dakikada Ankaferd grubunda tüm farelerde kanama durdu; chitosan grubunda sadece bir farede kanama durmadı. İlk dört dakikada kanamayı durdurmada Ankaferd, chitosan ile benzerdi fakat kontrol grubundan daha iyiydi; ayrıca chitosan kontrol grubuna benzerdi fakat p değeri anlamlılığa yakındı.

**Sonuç:**

Büyük arteryel kanamaların kontrolü için asıl tedavi cerrahi kanama kontrolüdür, fakat hastane dışı alanlarda Ankaferd ve chitosan içeren tamponlar kanama bölgesinde kullanılabilir. Araştırmamızın sonuçları daha geniş çalışmalarla desteklenmelidir. Ayrıca araştırmamızda sağlıklı fareler kullanılmıştır. Büyük arter kanamalı hipotansif ve hipovolemik olgulardaki sonuçları değerlendirmek için yeni çalışmalara ihtiyaç vardır.

## INTRODUCTION

Injury is the most frequent cause of death before the age of 45 years [1]. Major vascular injury is one of the major causes of death after trauma [2]. In the first assessment of trauma patients, in the circulation step, direct pressure should be applied to the sites of brisk external bleeding [2]. In this process, we need effective and rapid-acting materials to stop the bleeding.

Some of the procedures that can be used locally are direct pressure on bleeding, fibrin glues, microporous polysaccharide hemosphere (TraumaDEX®), poly-N-acetylglucosamine (Chitin®), microporous hydrogel forming polyacrylamide (BioHemostat®), chitosan linear polymer (Celox®), and oxidized cellulose (Bloodcare®) [3,4,5,6].

In this study, we compare the efficiencies of Ankaferd Blood Stopper® (ABS) and a chitosan linear polymer (Celox®) in an experimental rat model with femoral artery bleeding.

ABS is composed of folkloric herbal extracts that have been traditionally used in Anatolia as hemostatic agents (5), including Thymus vulgaris, Glycyrrhiza glabra, Vitis vinifera, Alpinia officinarum, and Urtica dioica. ABS, which contributes to the conventional methods to control bleeding, has been launched as a novel topical hemostatic agent for the management of visible hemorrhages [6,7,8,9,10]. ABS works by creating a protein network. It induces a very rapid (<1 s) formation of a cellular protein network, particularly including red blood cells and activated leukocytes within the whole blood sample, as well as within plasma and serum samples. It also induces the very rapid (<1 s) formation of vital erythroid aggregations as red blood cells clustered together to aggregate rapidly, thereby inducing a protein network formation. High-resolution scanning electron microscopy images accompanied by morphological analysis following the topical application of ABS revealed very rapid (<1 s) protein network formation within concurrent vital erythroid aggregation covering the classical coagulation cascade [11]. The overall hemostatic effects of ABS depend on the protein agglutination and polymerization modulating the erythroid aggregation and vascular endothelium. ABS also affects the distinct steps of cellular proliferation [12]. As an important advantage, ABS is also effective in patients with deficient primary and/or secondary hemostasis [13,14,15,16]. In addition to its anti-hemorrhagic properties, ABS has been shown to act as a topical biological response modifier [16]. All of these abilities not only make ABS an effective hemostatic agent, but they also confer anti-infective, anti-neoplastic, and healing modulator properties [17]. ABS has been used in a wide range of applications, from dental bleedings to gastrointestinal bleedings [18].

Chitosan (Celox®) is a non-toxic biological polysaccharide polymer of deacetylated chitin (poly [(1,4)-N-acetyl-D-glucose-2-amine)]) (19). It was approved by the United State Food and Drug Administration in June 2006 with ‘Generally Recognized as Safe’ status. The positive loaded NH3+ groups interact with negative loaded platelets and red blood cells, binding them with an ionic bond [20]. This causes the aggregation of platelets in the formation of thrombus. In vitro studies have shown its positive effects in wound healing on activation of polymorphonuclear neutrophils, macrophages, and fibroblasts [21,22,23]. Chitosan has antimicrobial activity against fungi and gram-positive and gram-negative bacteria that accelerates wound healing [22,24]. Celox® is a topical compound of chitosan that is used to stop bleeding of surface injuries [25].

## MATERIALS AND METHODS

The study was carried out with approval from the local experimental animals ethics committee (Ankara Numune Education and Research Hospital, 31.01.2011, protocol number: 2011/5). This study was performed in the Ankara Numune Education and Research Hospital animal laboratory and 30 male Wistar albino rats weighing 200-250 g were used. Rats were all fed with the same amount of feed and were fasted for 12 h before the study. Rats were divided into 3 groups as follows: in the control group (n=10), direct compression was applied to the bleeding without medication; in the Ankaferd group (n=10), direct compression was applied with ABS; and in the chitosan group (n=10), direct compression was applied with Celox®.

Before the experiment, xylazine hydrochloride and ketamine were used for anesthesia. At that time, arterial blood pressure monitorization was done with a KMA®250 monitor (Petaş, İstanbul, Turkey).

The right inguinal regions of the rats were wiped with Batticon and shaved, and the skin and subcutaneous tissues were cut into to reveal the femoral vein and artery. Bleeding was started with a total incision of the femoral artery and vein. Another person collected the accumulated blood with a sponge by pressing for 10 s. The sponge was removed and immediately the homeostatic material was applied (Celox® or ABS), and a constant 50 g of weight was put on this material. At this time, the timer was started. After the first minute, the bleeding was checked. If the bleeding had stopped, it was recorded as ‘bleeding stopped at the first minute’; if not, compression was continued with the same amount of material up to 2 min. After 2 min, the bleeding was checked. If the bleeding had stopped, it was recorded as ‘bleeding stopped at the second minute’; if not, compression was applied again with the same amount of material for 2 min. After these additional 2 min, the bleeding was checked. If the bleeding had stopped, it was recorded as ‘bleeding stopped at the fourth minute’. If the bleeding was still continuing, it was recorded as ‘unsuccessful’.

Before the rats were sacrificed under anesthesia with 100 mg/kg sodium thiopental (Pental Sodyum®, İ.E. Ulagay, İstanbul, Turkey), 3-mL blood samples were taken from the abdominal aorta in order to measure the levels of hemoglobin (Hb), hematocrit (Hct), coagulation parameters (activated partial thromboplastin time [APTT], prothrombin time [PT], and international normalized ratio [INR]), potassium (K), and calcium (Ca).

### Statistical Analysis

Statistical analysis was performed with SPSS 18.0 for Windows. Continuous variables were expressed as mean ± standard deviation and categorical parameters were given as numbers and percentages. For comparing continuous variables among more than 2 groups, the Kruskal-Wallis test was used. For comparison of categorical variables, Fisher’s exact test was used. All calculations were 2-tailed and p<0.05 was accepted as significant.

## RESULTS

Two rats were excluded from the study because their mean arterial pressures fell below 50 mmHg. Two new rats were added to the study in their place. At the end of the study, the rats that survived were sacrificed by 100 mg/kg intravenous sodium thiopental (Pental Sodyum®, İ.E. Ulagay).

Mean plasma K, Ca, Hb, Hct, and platelet levels; APTT, PT, and INR values; and weights of the groups are expressed in Table 1. These parameters were similar in all groups (p>0.05).

In the control group, 60% of the bleeding did not stop. In the Ankaferd group, the bleeding stopped in the first 4 min in all rats; only in 1 rat of the chitosan group did the bleeding not stop (Table 2). The bleeding did not stop in any rats in the first minute.

Among rats in which the bleeding stopped in the first 2 min, results with ABS were similar to those with chitosan (p=1.000) and to the control group (p=0.087), but the p-values were not statistically significant; chitosan results were also similar to those of the control group (p=0.211).

In stopping the bleeding in the first 4 min, ABS was similar to chitosan (p=1.000) and better than the control group (p=0.011); chitosan was similar to the control group (p=0.057), but the p-value was close to significance.

## DISCUSSION

Various procedures, such as direct compression, tourniquets, and clamps, are used to stop bleeding, but these methods do not always end in success. Homeostatic materials are now produced to deal with severe bleeding due to trauma. In this study, we compared 2 known homeostatic materials and direct compression without medication. There are limited studies that have compared ABS and Celox®. Aktop et al. evaluated hemostatic parameters and the early stages of healing potential with Celox® and ABS on soft tissue in warfarin-treated rats [26]. As in our study, they found no differences in hemostasis time, but they did find increased tissue factor values in the Celox®-treated group. Huri et al. also found no significant difference between Celox® and ABS hemostasis time [27]. However, tissue healing has been shown to be improved with ABS. Homeostatic agents are mainly used to stop venous and small arterial bleedings, but we used them for significant arterial bleedings and showed their efficiency even in major bleeding.

Ersoy et al. showed that microporous polysaccharide hemosphere shortens hemostasis time [3]. Hanks et al. compared the homeostatic efficiencies of fibrin glue and oxidized cellulose among patients who had undergone multiple surgical operations; they reported a shorter homeostatic time in the fibrin glue group, where the bleeding time was 1.6 min as opposed to 3.3 min with oxidized cellulose [28]. In our study, in the ABS group, the bleeding stopped in 40% of the rats in the first 2 min and in the remaining rats in the first 4 min. In this rat model with femoral bleeding, ABS was better than direct compression to stop the bleeding in the first 4 min (p=0.011). In 60% of the control group, the bleeding did not stop in the first 4 min, but in the ABS group, the bleeding stopped in the first 4 min in all rats.

Topical homeostatic agents have additional advantages. There are some studies reporting that they reduce secondary complications in some interventions; acidic forms have antibacterial and anticandidal effects and they accelerate wound healing [28,29].

## CONCLUSION

In conclusion, in the case of major bleeding, the main treatment is surgical bleeding control, but outside of the hospital, we have to use bleeding control procedures. Here we showed that ABS and chitosan are better than direct pressure on the bleeding region.

## Study Limitations

The results of this study should be supported with larger studies. Additionally, in our study, healthy rats were used. New studies are needed to evaluate the results of already hypovolemic and hypotensive subject groups in major artery bleeding.

## Ethics

Ethics Committee Approval: Ankara Numune Education and Research Hospital, 31.01.2011, protocol number: 2011/5, Informed Consent: N/N.

## Figures and Tables

**Table 1:**
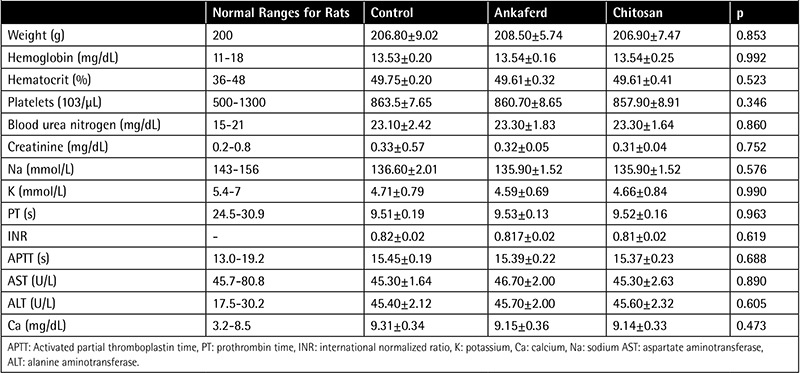
Weight and blood test results of the groups.

**Table 2:**
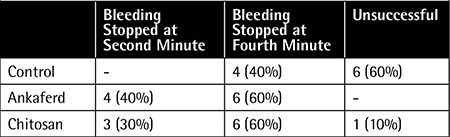
Homeostasis durations of the groups.
